# Distinct Signatures of Chromosomal Involvement in 59 251 Translocations Across 58 Tumor Types. A Novel Perspective

**DOI:** 10.1002/gcc.70053

**Published:** 2025-05-10

**Authors:** Felix Mitelman, Nils Mandahl

**Affiliations:** ^1^ Division of Clinical Genetics, Department of Laboratory Medicine Lund University Lund Sweden

**Keywords:** cancer, chromosomes, neoplasia, translocations

## Abstract

Chromosomal translocations are key events in cancer, driving oncogenesis by disrupting and deregulating critical genes. While specific tumor‐associated translocations are well studied, the frequencies and distributions of most remain unknown. Additionally, the role of chromosomal reshuffling in translocations has received little attention. This study presents data on the chromosomal involvement in 59 251 translocations reported in 58 tumor entities, including both benign and malignant tumors. Unlike studies focusing on tumor‐specific abnormalities identified at the chromosome band level, this study examines translocations at the chromosomal level, offering a novel perspective on their distribution. This broader approach aims to uncover patterns that do not emerge or are disregarded in studies limited to tumor‐specific aberrations. The resulting dataset provides a novel resource for deepening our understanding of the chromosomal origins of translocations in neoplasia. Comparisons of translocation frequency distributions among tumor types, when excluding the characteristic tumor‐associated translocations, revealed that the patterns of chromosomal involvement in translocations are largely unique to each tumor entity. Statistical analyses of 241 pairwise comparisons of translocation spectra within hematologic disorders, solid tumors, and between groups of hematologic malignancies and both benign and malignant solid tumors showed insignificant/very weak associations (*R* ^2^ ≤ 0.3) in 98% of the comparisons. The findings hence demonstrate that different tumor types are characterized by distinct chromosomal translocation signatures, strongly suggesting that most translocations encountered in tumor cells are not merely random events. Consequently, our study highlights the potential of rare translocations to serve as indicators of disease‐specific processes.

## 1 Introduction

Cytogenetic analyses of tumor cells have been instrumental in establishing that neoplasia, at the cellular level, is a genetic disease. Clonal chromosome abnormalities have been identified in more than 80 000 neoplasms, including malignant hematologic disorders, lymphomas, and both benign and malignant solid tumors, and characteristic chromosomal abnormalities have been detected in nearly all tumor types examined [1]. Among the various types of chromosome abnormalities observed, balanced structural rearrangements—particularly reciprocal translocations—have emerged as especially significant. A growing number of recurrent, specific, and even pathognomonic translocations have been identified across various hematologic malignancies and solid tumors [2, 3, 4]. Molecular characterization of these translocations has provided critical insights into cancer biology. Genomic analyses have revealed that nearly all balanced translocations generate gene fusions at their breakpoints, leading either to deregulation—most often overexpression—of a gene at one of the breakpoints or to the formation of a hybrid gene through the fusion of gene segments from each breakpoint [2, 3, 5]. There is overwhelming evidence that these rearrangements drive cancer development.

The discovery of cancer‐associated gene fusions, resulting in either chimeric proteins or aberrant expression of one or both partner genes, has revolutionized the search for cancer‐causing genes and remains a highly successful approach [5]. However, little attention has been given to the potential role of chromosomal reshuffling in translocations. Chromosomes are not merely carriers of gene sequences but evolutionarily conserved structural entities [6, 7, 8, 9]. They are organized into distinct three‐dimensional territories occupying varying locations within the nucleus depending on cell type and differentiation stage. These territories can move, intermingle, and form chromatin loops that facilitate interactions between genes from different chromosomes within transcriptional hubs. Given the essential role of the higher‐order genome organization, it is reasonable to assume that translocations that disrupt this territorial structure—relocating chromosomal segments to new nuclear environments with distinct transcriptional and regulatory influences—may have far‐reaching consequences beyond the gene rearrangements at breakpoints.

This study analyzes 59 251 non‐homologous translocations across a diverse range of benign and malignant tumors. By comparing the frequency distributions of all possible translocation types, this large‐scale analysis reveals that distinct tumor types exhibit unique chromosomal translocation signatures. These findings strongly suggest that most translocations in tumor cells are not random events. Consequently, our study highlights the potential of rare translocations to serve as indicators of disease‐specific processes.

## 2 Materials and Methods

Clonal chromosome abnormalities were extracted from the Mitelman Database of Chromosome Aberrations and Gene Fusions in Cancer [1]. When queried on 29 August 2024, the database contained 83 272 cases of neoplastic disorders that could be unequivocally classified as benign or malignant, including 8480 unpublished cases from our laboratory.

When assessing translocation frequencies in individual tumor entities, only those entities in which at least 100 cases were available and in which at least 100 translocations had been identified were considered, leaving 68 507 cases in 58 tumor entities for evaluation. Translocation frequencies were also assessed in the following 11 main tumor groups comprising 76 264 cases: Acute myeloid leukemia (AML, *n* = 23 361), myelodysplastic syndromes/myeloproliferative disease/chronic myeloproliferative disorders (MDPD, *n* = 8794), acute lymphoblastic leukemia (ALL, *n* = 12 743), plasma cell neoplasms (PCN, *n* = 2590), mature B‐cell neoplasms (B‐ML, *n* = 10 405), mature T‐ and NK‐cell neoplasms (T/NK‐ML, *n* = 1467), benign epithelial tumors (BET, *n* = 1189), malignant epithelial tumors (MET, *n* = 6523), malignant neuroectodermal tumors (MNET, *n* = 1664), benign mesenchymal tumors (BMT, *n* = 3621), and malignant mesenchymal tumors (MMT, *n* = 3907). Numbers of cases included in individual tumor entities and tumor groups are presented in Table.

All translocations irrespective of their band‐level involvement were ascertained. Only two‐way translocations were considered, and those involving homologous chromosomes and the Y chromosome were excluded, leaving a total of 59 251 translocations for detailed analysis. The frequencies of each of the 253 different translocation types, that is, t(1;2) through t(X;22), were calculated separately for each tumor entity and tumor group.

Coefficients of determination (*R* ^2^) were computed to quantify the strength of association in translocation frequency patterns across neoplasm pairs, using data from the 28 most extensively characterized tumor entities, comprising 12 hematologic malignancies and 16 solid tumor types, and the 11 principal tumor groups. Acute myeloid leukemias, classified according to the FAB system [10], were grouped into the following related categories: myeloblastic leukemias (M0–M2), acute promyelocytic leukemia (M3), myelomonocytic and monocytic leukemias (M4–M5), and erythroid and megakaryoblastic leukemias (M6–M7). To minimize the impact of tumor‐associated characteristic translocations, those accounting for more than 10% of all observed translocations—such as t(9;22) in chronic myeloid leukemia and t(X;18) in synovial sarcoma—were excluded. Statistical thresholds were defined as follows: insignificant/very weak (0.0–0.3), weak/moderate (0.4–0.6), and strong/very strong (0.7–1.0).

## 3 Results

The results are presented in Tables S2 and S3. All abbreviations used in the text and tables are provided in Table S1. Table S2 shows the frequencies of all translocation types in absolute numbers (S2A) and as percentages (S2B) in 32 hematologic disorders, and S2C and S2D show the corresponding data for 26 solid tumor types. Table S3 summarizes frequencies across the 11 tumor groups, both in absolute numbers (S3A) and as percentages (S3B).

All 253 possible translocation types were identified in at least one of the 58 tumor entities, and consequently also in at least one of the 11 tumor groups. In all tumor entities, there is a strong association between the numbers of cases investigated and the numbers of translocations found (*R* ^2^ > 0.8).

The total numbers of translocations as well as frequencies of translocations occurring in 0%, < 1%, 1%–≤ 10% and > 10% are presented in S2B, S2D, and S3B. Apart from the few well‐known characteristic translocations, the vast majority were rare or exceedingly rare. Translocations occurring at frequencies below 1% accounted for 79.8% to 99.2% of all translocations in hematologic disorders, 82.2% to 98.8% in solid tumors, and 88.5% to 96.0% across the 11 tumor groups. Furthermore, 12 of the 32 hematologic disorders and 15 of the 26 solid tumors had no translocations seen at frequencies above 10%. The 24 translocations found in > 10% are shown in Table S4.

**FIGURE 1:**
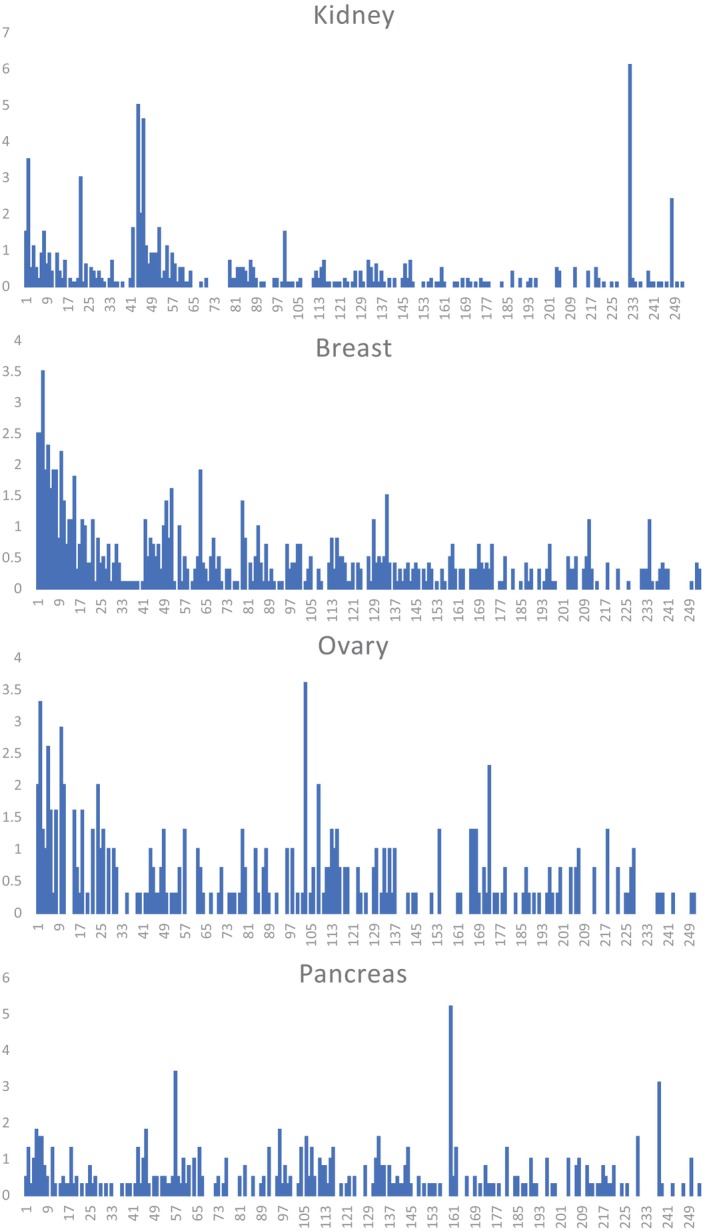
Frequencies (%) of all 253 translocations from t(1;2) to t(X;22) showing the different translocation signatures for adenocarcinomas of the kidney, breast, ovary, and pancreas. The figures on the *X*‐axis refer to translocation types 1–253 shown in Table S2D. The *Y*‐axis shows the percentage frequencies.

*R* ^2^ values for 241 pairwise comparisons of translocation spectra among 12 hematologic malignancies, 16 solid tumor types, and 11 tumor groups are shown in Table 1. The *R* ^2^ values revealed distinct translocation signatures for each tumor entity and each tumor group. Among hematologic disorders (Table 1a), 65 of the 66 (98%) comparisons showed *R* ^2^ values ≤ 0.3 (< 0.1 in 54). Among solid tumors (Table 1b) 118 of 120 (98%) comparisons showed *R* ^2^ values ≤ 0.3 (< 0.1 in 91). Also, when comparing tumor groups encompassing both hematologic disorders and solid tumors (Table 1c) similar results were obtained, that is, *R* ^2^ ≤ 0.3 in 53 of 55 (96%) comparisons (< 0.1 in 38). In total, thus, 236 of the 241 comparisons (98%) showed *R* ^2^ values ≤ 0.3, that is, insignificant/very weak associations. Examples of characteristic signatures for adenocarcinomas of the kidney, breast, ovary, and pancreas are illustrated in Figure 1.

**TABLE 1:** *R* ^2^ values in pair‐wise comparisons of translocation spectra.

a. Hematologic disorders											
	AML M3	AML M4–M5	AML M6–M7	MDS	ALL	MM	CLL	FL	DLBL	BL	T/NK‐ML
AML M0–M2	0.089	0.334	0.213	0.192	0.029	0.010	< 0.001	0.004	0.004	0.001	0.010
AML M3		0.119	0.066	0.057	0.002	0.016	< 0.001	0.002	0.001	< 0.001	0.002
AML M4–M5			0.048	0.084	0.033	0.019	< 0.001	0.002	0.002	< 0.001	0.013
AML M6–M7				0.121	0.009	0.067	< 0.001	0.024	0.009	< 0.001	0.021
MDS					0.006	0.097	< 0.001	0.070	0.030	< 0.001	0.049
ALL						0.077	0.032	0.021	0.047	0.020	0.015
MM							0.015	0.236	0.206	0.105	0.056
CLL								0.048	0.131	0.203	0.021
FL									0.599	0.075	0.079
DLBL										0.129	0.071
BL											0.005

b. Solid tumors															
	BCA	OCA	HNCA	LCA	PCA	Astcyt	MMEL	Lip	Lei	SA lip	SA lm	SA rm	SA syn	SA ost	SA Ew
KCA	0.123	0.055	0.141	0.094	0.018	0.022	0.062	0.003	0.013	0.017	0.040	0.012	0.094	0.054	< 0.001
BCA		0.275	0.400	0.309	0.028	0.122	0.286	0.049	0.132	0.143	0.096	0.165	0.077	0.138	0.035
OCA			0.184	0.300	0.054	0.090	0.186	0.033	0.123	0.058	0.077	0.078	0.065	0.095	0.021
HN CA				0.235	0.008	0.153	0.364	0.021	0.004	0.070	0.116	0.150	0.083	0.202	0.018
LCA					0.049	0.062	0.149	0.016	0.071	0.061	0.095	0.065	0.082	0.084	0.015
PCA						0.011	0.008	0.004	0.023	0.001	0.038	0.004	0.010	0.019	< 0.001
Astcyt							0.103	0.003	0.051	0.029	0.026	0.088	0.016	0.091	0.050
MMEL								0.037	0.135	0.086	0.077	0.131	0.088	0.137	0.061
Lip									0.149	0.304	0.019	< 0.001	0.031	0.012	< 0.001
Lei										0.094	0.058	0.044	0.013	0.068	< 0.001
SA lip											0.008	0.036	0.053	0.021	0.094
SA lm												0.046	0.030	0.056	0.002
SA rm													0.006	0.154	0.116
SA syn														0.004	0.005
SA ost															0.016

c. Tumor groups										
	MDPD	ALL	PCN	B‐ML	T/NK‐ML	BET	MET	MNET	BMT	MMT
AML	0.162	0.039	0.027	0.002	0.026	0.076	0.094	0.068	0.035	0.006
MDPD		0.015	0.132	0.088	0.061	0.185	0.297	0.183	0.169	0.008
ALL			0.056	0.017	0.034	0.007	0.012	0.032	0.009	< 0.001
PCN				0.235	0.064	0.069	0.281	0.386	0.049	0.031
B‐ML					0.109	0.020	0.052	0.061	0.017	0.001
T/NK‐ML						0.053	0.124	0.125	0.042	0.004
BET							0.180	0.120	0.107	0.004
MET								0.405	0.090	0.012
MNET									0.102	0.081
BMT										0.005

Abbreviations: ALL = acute lymphoblastic leukemia; AML = acute myeloid leukemia (all FAB subtypes); AML M0‐M7 = acute myeloid leukemia FAB subtypes; Astcyt = astrocytoma III‐IV; BCA = breast cancer; BET = benign epithelial tumors; BL = Burkitt lymphoma; B‐ML = B‐cell malignant lymphoma; BMT = benign mesenchymal tumors; CLL = chronic lymphocytic leukemia; DLBL = diffuse large B‐cell lymphoma; FL = follicular lymphoma; HNCA = head and neck cancer; KCA = kidney cancer; LCA = lung cancer; Lei = leiomyoma; Lip = lipoma; MDPD = myelodysplastic syndromes/myeloproliferative disease/chronic myeloproliferative disorders; MDS = myelodysplastic syndromes; MET = malignant epithelial tumors; MM = multiple myeloma; MMEL = malignant melanoma; MNET = malignant neuroepithelial tumors; OCA = ovarian cancer; PCA = pancreatic cancer; PCN = plasma cell neoplasms; SA Ew = Ewing sarcoma; SA lip = liposarcoma; SA lm = leiomyosarcoma; SA ost = osteosarcoma; SA rm. = rhabdomyosarcoma; SA syn = synovial sarcoma; T/NK‐ML = T/NK‐cell malignant lymphoma.

## 4 Discussion

Chromosome banding analyses have been instrumental in identifying a large number of characteristic, specific, and even pathognomonic translocations in practically all tumor entities studied in sufficient numbers [1, 2, 3, 4]. Such tumor‐specific translocations, however, constitute only a small minority of all observed rearrangements reported in neoplastic cells [1]. We have recently presented and cataloged a compilation of breakpoint locations in all known translocations and fusion genes in cancer [4]. Characterization of the breakpoints demonstrated that despite the fundamentally different resolution levels in studies at the cytogenetic and molecular genetic levels, the results were concordant. Giemsa‐negative bands were predominantly involved in both translocations and fusion genes, indicating that they may be mediated by some underlying mechanism that either favors the origin or provides advantage for the recombination of such bands. By shifting the focus from the chromosome band level to a broader chromosomal level, we now provide a novel perspective on the distribution of translocations. Cataloging the full range of translocations from common to exceedingly rare events may help to identify patterns that are not apparent in studies focusing on tumor‐specific aberrations. This extensive dataset, encompassing 59 251 translocations in 58 tumor entities, will hopefully provide a valuable resource to gain a deeper insight into the landscape of the chromosomal origin of translocations in both benign and malignant neoplasms, potentially providing a further understanding of principles that guide tumor genome organization and evolution.

**FIGURE 2:**
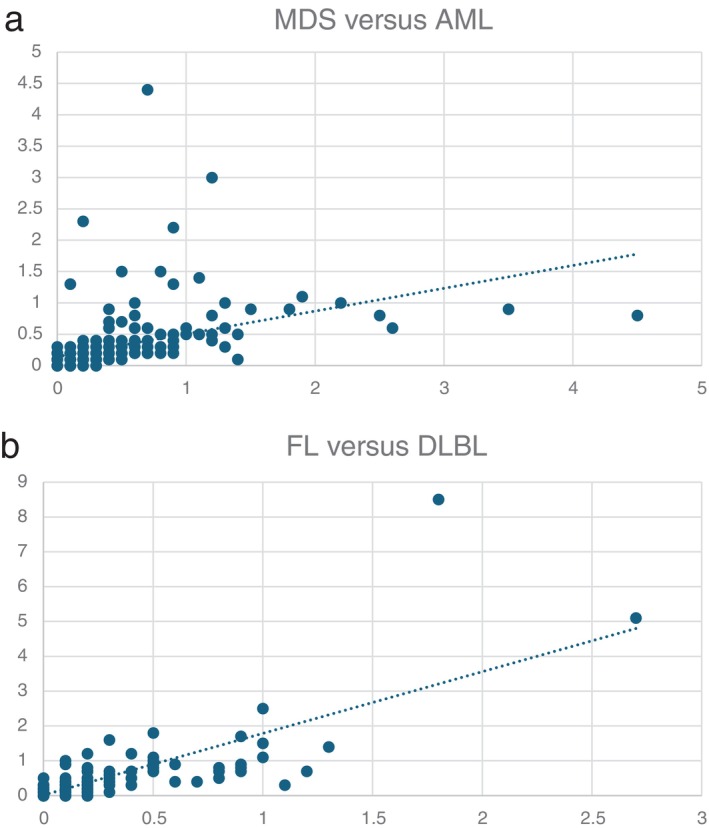
Pairwise comparisons of translocation spectra in (a) MDS versus AML (*R* ^2^ = 0.128) and (b) FL versus DLBL (*R* ^2^ = 0.599).

Comparisons of translocation distributions among tumor types were conducted to discern similarities and differences across various tumor entities. Since characteristic translocations that may dominate certain tumor entities could severely skew and override other possible differences, such aberrations (defined as those present in > 10% of any tumor entity) were excluded from these analyses. This approach should, at least to some extent, illuminate the role played by the majority of the individually rare translocations seen in most tumor types. In fact, we found that, depending on tumor type, 80%–90% of all translocations occur at frequencies below 1%. The distribution of these rare events may shed light on a long‐standing question of whether non‐recurrent abnormalities in tumors represent irrelevant chance events or may be significant for tumor development. The comparisons of the translocation frequencies using the present approach revealed significant differences in the translocation distributions among tumor types. Thus, *R* ^2^ values for 241pair‐wise comparisons within hematologic disorders and solid tumors, as well as between groups of hematologic malignancies and both benign and malignant solid tumors, were found to be insignificant/very weak in 98% of comparisons (Table 1). The findings hence demonstrate that different tumor types are characterized by different chromosomal translocation signatures. The divergent translocation spectra are apparent not only between clinically and genetically distinctly different tumor types, such as AML versus ALL, mesenchymal versus epithelial tumors, and MET arising in different organ systems. Even closely related conditions with overlapping clinico‐pathologic features exhibit distinct translocation signatures. The most striking example is the situation in MDS and AML. Both diseases originate from abnormal myeloid progenitor cells in the bone marrow and share common genetic mutations (e.g., *TET2*, *ASXL1*, *EZH2*, and *DNMT3A*), as well as monosomy 7 and trisomy 8, the two most frequent cytogenetic aberrations in both disorders [11, 12]. Moreover, progression of MDS to AML is well documented, supporting the view that MDS and AML may represent a biological continuum along a spectrum of myeloid neoplasms rather than entirely distinct diseases. The different translocation spectra when comparing all AML subtypes with MDS (Figure 2a) indicate that they may influence disease progression and phenotype. In this context, it is of interest that the presence or absence of monosomy 7 and trisomy 8, despite being common to both MDS and AML (found in 27% and 34% of cases, respectively), had no significant impact on the translocation patterns when MDS cases with −7 or +8 and AML cases with these abnormalities were compared (data not shown). These findings may be taken as further support that the translocation signatures indeed play an important role in shaping the leukemia phenotype.

The only comparable situation of closely related tumor types that could be compared in this study involves follicular lymphoma (FL) and diffuse large B‐cell lymphoma (DLBL). These two malignant lymphoma subtypes share a common B‐cell origin, overlapping genetic abnormalities, and the potential for progression from FL to DLBL [13]. Among all tumor comparisons in this study, the highest *R* ^2^ value (0.6) was found when comparing FL with DLBL (Figure 2b), indicating a borderline association between moderate and strong correlation. This finding thus agrees with the generally accepted view that the two disease types are indeed closely related and provides further validation of the assumption that the translocation signatures identified in this study can reflect biologically meaningful information as regards relationships between tumor types.

An important point that warrants discussion is the choice of the > 10% cut‐off level used in calculating associations among tumor types. This threshold was selected to characterize rare translocations as accurately as possible while minimizing the influence of well‐known, tumor‐specific abnormalities. The 10% level was deemed reasonable as it encompassed practically all the well‐documented, but relatively few, characteristic translocations associated with specific tumor types. One could argue that this threshold may be too high to effectively capture the possibly stochastic, non‐recurrent events that were central to our investigation. However, it is important to note that 90% of the translocations were found at frequencies below 1%, and only a small fraction (9.5%) fell within the 1%–10% range. This distribution suggests that the analysis was primarily focused on rare events, as intended. To assess whether this chosen threshold introduced any systematic bias or error in our comparisons, we performed additional analyses using progressively stricter cut‐off levels of 5%, 2%, and 1%. We selected five representative associations with *R* ^2^ values ranging from 0.029 to 0.599 for this evaluation: MDS versus AML, AML M0–M2 versus AML M4–M5, AML M0–M2 versus ALL, FL versus DLBL, and breast carcinoma versus head and neck carcinoma. Across all comparisons, the *R* ^2^ values changed only marginally when lower cut‐off thresholds were applied. This consistency indicates that the 10% cut‐off adequately represents the underlying variation in translocation distributions without introducing significant distortion or bias. Thus, we conclude that our selected threshold was appropriate for capturing the rare translocations while minimizing the impact of common tumor‐specific abnormalities, ultimately supporting the reliability of our findings.

**FIGURE 3:**
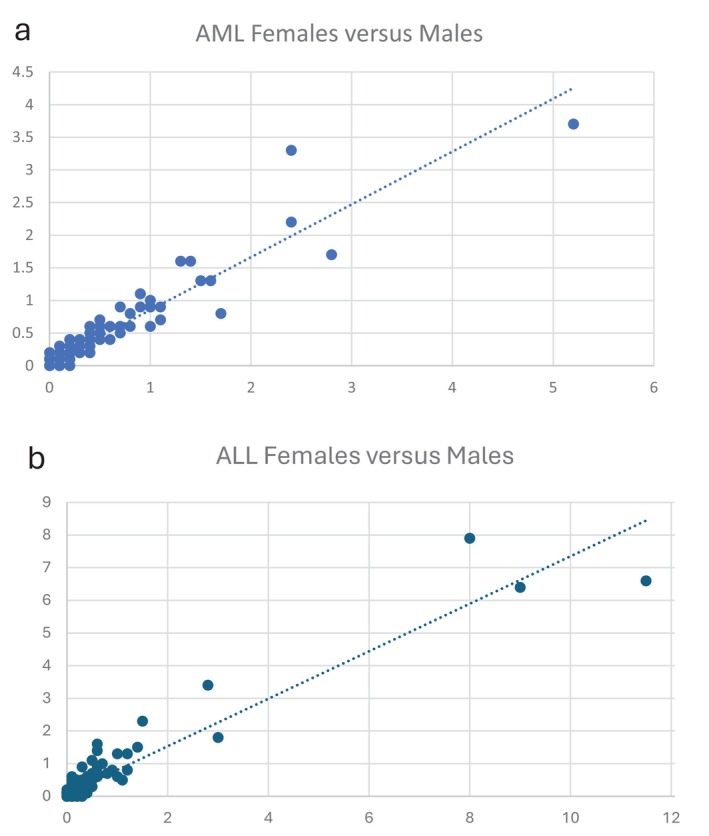
Pairwise comparisons of translocation spectra in (a) AML females versus males (*R* ^2^ = 0.880) and (b) ALL females versus males (*R* ^2^ = 0.899).

A decisive argument that the rare translocations we identified are not merely random events is the striking concordance of translocation signatures between females and males within the same tumor types. Figure 3 (data presented in Table S3C) illustrates this comparison in the two most extensively studied neoplastic disorders: AML and ALL. As shown, the distribution patterns of these rare translocations are nearly identical between sexes in both leukemia types, with *R* ^2^ values of 0.9 in each case. Such a high degree of similarity is incompatible with the interpretation that these events occur randomly, effectively ruling out that explanation.

The central question to address is what the rare translocations signify. The striking feature of the chromosomal aberrations in neoplasia is their enormous heterogeneity. Practically all kinds of structural and numerical abnormalities have been reported, and very often several changes are present simultaneously [1]. Unquestionably, some are without doubt of pathogenetic significance in that their molecular consequences have been clarified and shown to be causally important in the tumorigenic process either by oncogene activation or tumor suppressor gene inactivation [2]. Such primary aberrations may be found as the sole karyotypic change and are often specifically associated with particular tumor types. The great majority of chromosomal abnormalities are, however, very rarely or never seen as the sole anomaly [1]. They may, on the other hand, be so numerous as to completely dominate the karyotypic picture. Such secondary abnormalities may nevertheless demonstrate nonrandom features with distribution patterns that appear to be dependent on the type of the primary abnormality present, for example, the stepwise accumulation of +8, i(17)(q10), +19, and +der(22) in CML with t(9;22)(q34;q11) [14]. Similar acquisition of characteristic secondary aberrations has been reported in several tumor types, for example, +8 in AML with t(8;21)(q22;q22) and myxoid liposarcoma with t(12;16) (q13;p11), der(16)t(1;16)(q11–12;q11) in Ewing sarcoma with t(11;22)(q24;q12), and +1q abnormalities in multiple myeloma with t(4;14) (p16;q32) [1, 2]. The nonrandom nature of secondary aberrations associated with primary cytogenetic changes has been discussed extensively [15]. In summary, they may be explained by two main mechanisms: primary abnormalities may drive selective pressures that favor the emergence of specific secondary aberrations, thereby promoting tumor progression, or primary abnormalities may restrict the range of possible secondary changes, limiting the types of abnormalities that can develop. In many cases, these mechanisms may act in combination. Given the vast amount of data on chromosome changes in cancer—currently almost 50 000 unique cytogenetic abnormalities have been reported [1]—it is surprising how few well‐characterized secondary aberrations have been identified. The results of the present study, which aimed to capture rare clonal aberrations, indicate that also these seemingly stochastic abnormalities are, in fact, nonrandom and exhibit distinct aberration patterns in different tumor types. This leads to two key possibilities regarding their origin: (1) The aberrations may be secondary to a primary change. This is obvious in all instances where they co‐exist with a well‐known primary cytogenetic change, like t(8;21), t(15;17) or t(9;22). In most instances, however, the primary change remains unidentified, making it impossible to formally categorize an abnormality as secondary. (2) The abnormalities may represent primary changes that are so rare that they have not yet been recognized. Their nature as primary abnormalities will become apparent when more cases have been studied and the corresponding cancer phenotypes are better understood.

In conclusion, this study has uncovered numerous translocations that are either potentially primary and pathogenetically significant or secondary and evolutionary important, with distinct patterns that vary across tumor types. These results contradict the common belief that rare and diverse abnormalities are mere noise and therefore biologically irrelevant. The observed diversity is, in fact, consistent with our understanding of tumor biology. Tumors originating in different tissues exhibit distinct regulatory mechanisms, signaling pathways, mutational landscapes, and selective pressures, all of which drive unique genetic adaptations within their respective microenvironments [16, 17, 18, 19].

## Ethics Statement

The authors have nothing to report.

## Conflicts of Interest

The authors declare no conflicts of interest.

## Supporting information


Table S1.

Table S2.

Table S3.

Table S4.

## Data Availability

The data on which this study was based are freely available at https://mitelmandatabase.isb‐cgc.org. Data sources and handling of the publicly available data sets used in this study are described in the Materials and Methods section and in Tables [Link], [Link], [Link], [Link].
