# Supplementary material for: Distinct Signatures of Chromosomal Involvement in 59 251 Translocations Across 58 Tumor Types. A Novel Perspective
Source: Genes Chromosomes Cancer. 2025 May 10;64(5):e70053. doi: 10.1002/gcc.70053 (PMC12065015; doi:10.1002/gcc.70053)
Supplement: Supplementary file 4 — Table S4. [file GCC-64-e70053-s001.docx]

Supplementary Table 4. Translocations found in >10% in 33 tumor entities. For abbreviations, see Table S1

| **Translocation type** | **Tumor entity** |
| --- | --- |
| t(1;16) | Wilms |
| t(1;17) | Astcyt II-IV |
| t(1;22) | AML M7 |
| t(2;5) | ALCL-S |
| t(2;13) | SA rhabd |
| t(3;5) | Kidney CA |
| t(3;8) | BET |
| t(3;12) | Lip |
| t(5;12) | MDPD |
| (6;14) | Chondr ham |
| t(8;14) | B-ML NOS, BL |
| t(8;21) | AML NOS, AML M1, AML M2 |
| t(9;11) | AML M5 |
| t(9;22) | CML, ALL |
| t(11;14) | MM, PCL, MCL |
| t(11;18) | MALT |
| t(11;22) | SA Ewing |
| t(12;14) | Lei, Chondr ham |
| t(12;16) | SA lip |
| t(14;18) | B-ML NOS, FL, DLBL |
| t(14;19) | NMZL |
| t(15;17) | AML M3 |
| t(X;14) | T-PLL |
| t(X;18) | SA syn |
